# Enhanced Susceptibility of Nasal Polyp Tissues to Avian and Human Influenza Viruses

**DOI:** 10.1371/journal.pone.0012973

**Published:** 2010-09-24

**Authors:** Ornpreya Suptawiwat, Pongsakorn Tantilipikorn, Chompunuch Boonarkart, Jate Lumyongsatien, Mongkol Uiprasertkul, Pilaipan Puthavathana, Prasert Auewarakul

**Affiliations:** 1 Department of Microbiology, Faculty of Medicine Siriraj Hospital, Mahidol University, Bangkok, Thailand; 2 Department of Oto-Rhino-Laryngology, Faculty of Medicine Siriraj Hospital, Mahidol University, Bangkok, Thailand; 3 Department of Pathology, Faculty of Medicine Siriraj Hospital, Mahidol University, Bangkok, Thailand; University of Georgia, United States of America

## Abstract

**Background:**

Influenza viruses bind and infect respiratory epithelial cells through sialic acid on cell surface. Differential preference to sialic acid types contributes to host- and tissue-tropism of avian and seasonal influenza viruses. Although the highly pathogenic avian influenza virus H5N1 can infect and cause severe diseases in humans, it is not efficient in infecting human upper respiratory tract. This is because of the scarcity of its receptor, α2,3-linked sialic acid, in human upper airway. Expression of sialic acid can be influenced by various factors including inflammatory process. Allergic rhinitis and nasal polyp are common inflammatory conditions of nasal mucosa and may affect expression of the sialic acid and susceptibility to influenza infection.

**Methodology/Principal Finding:**

To test this hypothesis, we detected α2,3- and α2,6-linked sialic acid in human nasal polyp and normal nasal mucosal tissues by lectin staining and infected explants of those tissues with avian influenza viruses H5N1 and seasonal influenza viruses. We show here that mucosal surface of nasal polyp expressed higher level of α2,3- and α2,6-linked sialic acid than normal nasal mucosa. Accordingly, both H5N1 avian influenza viruses and seasonal influenza viruses replicated more efficiently in nasal polyp tissues explants.

**Conclusions/Significance:**

Our data suggest a role of nasal inflammatory conditions in susceptibility to influenza infection, especially by avian influenza viruses, which is generally inefficient in infecting human upper airway. The increased receptor expression may contribute to increased susceptibility in some individuals. This may contribute to the gradual adaptation of the virus to human population.

## Introduction

The viral surface protein, hemagglutinin, of influenza viruses can bind to various types of sialic acid molecules on cell surface glycans [1]. The sialic acid serves as main receptor for influenza virus binding and entry into target cells. Preference to the two major receptor types, α2,3- and α2,6-linked sialic acid, is a pivotal difference between avian and human influenza viruses [2]. The main target cell of H5N1 avian influenza viruses (AIVs) in humans is type II alveolar epithelial cells [3], which express the α2,3-linked sialic acid abundantly [4]. In contrast to alveoli, epithelia of human upper airway express mainly α2,6-linked sialic acid and lack α2,3-linked sialic acid [4]. Using α2,3-linked sialic acid, AIVs including the highly pathogenic H5N1 viruses, do not infect human upper airway efficiently. However, in vitro explants of tissues from human nasopharynx and tonsil have been shown to be susceptible to infection by H5N1 AIVs [5]. It is not known how H5N1 AIV establishes infection in humans. Possibilities are direct invasion of lung via aerosols and minimal infection in upper airway with spreading into lung via minor aspiration. The presence of virus in nasopharyngeal aspirates and throat swabs suggest that infection of upper airway exists and may precede lung infection.

Expression of sialic acid on cell surface can be affected by multiple factors, including cellular differentiation, oncogenesis, and inflammation [6], [7]. Cell surface sialic acid was shown to affect histamine release in allergic conditions [8]. Sialic acid content in mucin produced by nasal mucosa can be altered by allergic reaction [9]. Nasal polyp is a common condition caused by chronic allergic or inflammatory process. We asked whether mucosal surface of nasal polyps contained an altered level of sialic acid. Because availability of suitable receptor can determine efficiency of infection, altered levels of cell surface sialic acid may affect the susceptibility to influenza viruses, especially for α2,3-linked sialic acid, which is scarce in upper airway and if upregulated may enhance susceptibility to H5N1 AIV.

## Materials and Methods

### Nasal polyp and mucosal tissues

Six nasal polyposis patients schedule for turbinate reduction procedures (turbinoplasties) were recruited for the study. All polyposis patients had the skin prick test positive for the common allergens in Thailand. For the comparison group, four patients with the diagnosis of inferior turbinate hypertrophy and scheduled for partial inferior turbinectomies were recruited. Informed consents were signed by the patients and the study was approved by the Institutional Review Board of Faculty of Medicine Siriraj Hospital. During the surgeries (polypectomies or turbinoplasties), small pieces of tissues (polyps vs. mucosa) were cut and immediately sent to the laboratory.

### Tissue culture and viral infection

The tissues were extensively washed and immediately placed into culture medium (F-12K nutrient mixture with L-glutamine, and antibiotics) (Gibco BRL, USA) in 24-well tissue culture plates. The tissues were infected with 1×10^6^ tissue culture infectious doses 50% (TCID_50_) of influenza A viruses of subtypes H5N1 [A/Thailand/3 (SP-83)/04] or H1N1 [A/Thailand/Siriraj-3/06 (H1N1)] within three hours after collection. After two hours infection the unattached virus was removed by washing twice with PBS. The tissues were incubated at 37°C in 5% CO_2_ incubator for 0, 20, 24 and 48 hours before the supernatants were collected for virus titrating by plaque assay. The tissues were then fixed in 10% neutral buffered formalin and processed for histological sections.

### Lectin staining

Tissue section were deparaffinized with xylene for 5 minutes then sequentially hydrated with 100, 95 and 80% alcohol for 5 minutes at each step. The tissues were then blocked for non-specific binding with 3% bovine serum albumin (Sigma, USA) in phosphate buffer saline (PBS) for 1 hour. After discarding blocking solution, tissues were incubated with 1 µg of FITC-conjugated Maackia amurensis I lectin (MAA I) or Sambucus nigra lectin SNA (Vector Laboratories, USA) in blocking solution for 1 hour at room temperature, then washed twice with PBS and finally counterstained with Evan's blue for 10 minutes. The slides were mounted and visualized under fluorescence microscopy. In some experiments, fresh tissue were pre-digested with neuraminidase from *Clostridium perfringens* (Sigma, USA) at a concentration of 1U/ml in PBS for 1 hour at 37°C before performing paraffin section and the lectin staining in order to confirm the specificity. Six lectin-stained sections of nasal polyps and nasal turbinates from six patients were counted for epithelial cells with positive and negative staining in three fields of the slide to calculate average percentages of sialic acid-positive cells. About 200 cells were counted in each slide.

### RNA extraction & Real time RT-PCR

Fresh tissues were cut into small pieces with a sterile surgical blade. Total RNA was isolated from small quantities of tissue (1 to 10 mg) using 800 µl TRIZOL reagent (Invitrogen, USA) and then purified using Qiagen RNAeasy kit according to the manufacturer's instructions. One microgram of total RNA was reverse transcribed with avian myeloblastosis virus reverse transcriptase (Promega, USA) and a random hexamer. The amplification was then performed in a Sybr green dye detection format (LightCycler; Roche, USA). The amplification reactions contained 1×LightCycler Fast Start DNA Master Sybr Green dye I (LightCycler; Roche, USA) and 0.4 mM of each forward and reverse primer. Melting-curve analyses were performed from 65°C to 95°C. The following primers were used to detect the expression of specific genes: ST3GAL1 forward and reverse [10]; ST3GAL4 forward and reverse [11]; E14134 and E14135 for glyceraldehyde-3-phosphate dehydrogenase (GAPDH) mRNA [12]. Reactions were performed in triplicates. All quantitations (threshold cycle [C*_T_*] values) were normalized to that of GAPDH to generate ΔC*_T_*, and the difference between the ΔC*_T_* value of the nasal polyp tissue and that of the reference (nasal mucosa tissue) was calculated as ΔΔC_T_. The relative level of gene expression was expressed as 2^−ΔΔCT^.

### Detection of viral infection in vitro

Paraffin-embedded, infected and non-infected control tissue sections were deparaffinized and rehydrated. Endogenous peroxidase activity was blocked by incubating the slides in 3% hydrogen peroxide for 15 minutes at room temperature. Tissue sections were treated with pre-warmed (37°C) 200 µg/mL proteinase K in TE buffer (50 mM Tris-HCl, 10 mM EDTA, pH 8.0) for 15 minutes at 37°C. A biotinylated anti-sense probe for in situ hybridization was prepared by in vitro transcription from a full-length viral nucleoprotein clone. Tissue sections and probes were heated in hybridization buffer (50% formamide, 3X SSC, 1X Denhardt's solution, 200 µg/mL Yeast tRNA, 50 mM sodium phosphate pH 7.4, and 1 mg/mL of dextran sulfate in diethyl pyrocarbonate-treated water) for 10 minutes at 90°C, placed on ice for 10 minutes, then mixed and left to hybridize overnight at 37°C in humidified chamber. The hybridization signal was developed with conjugated Streptavidin-Horseradish Peroxidase (PIERCE, USA) and diaminobenzidine. The sections were then counterstained with hematoxylin.

### Statistical analysis

Correlations between percentages of lectin-stained cells and viral titers produced the same tissues were determined using Pearson correlation analysis and linear regression analysis. All statistical computations were performed using SPSS software (version 16.0, SPSS Inc., Chicago, IL).

## Results

### Expression of sialic acid on nasal polyps

**Figure 1:**
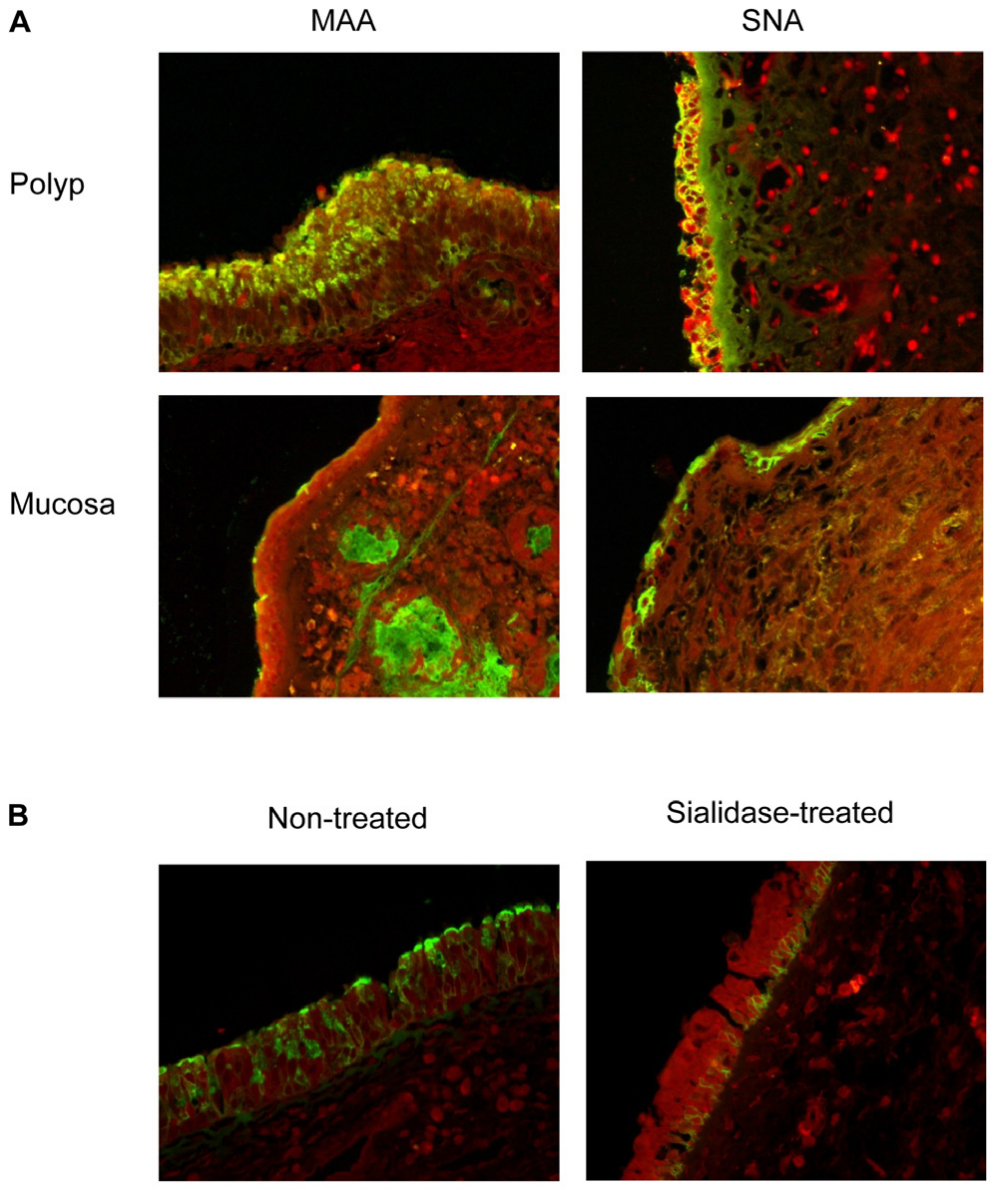
Representative micrographs of nasal polyp and nasal turbinate mucosa showing distribution of α2,3- and α2,6-sialic acid. Tissue sections were stained with FITC conjugated lectin MAA I (left panel) or SNA (right panel), specific toward α2,3- or α2,6-linked sialic acid, respectively (a). To confirm the specificity and the presence of α2,3-linked sialic acid on nasal polyp, tissues were digested with 1U/ml of sialidase before MAA I staining. The sialidase-treated tissue (right) lost the staining signal on the apical surface, while non-treated section was positive (left) (b).

Initially four nasal polyp and four nasal turbinate mucosa samples were examined and tissue sections were stained with the lectins. Histological examination showed normal intact nasal mucosa in the turbinate specimens and submucosal infiltration of eosinophils, lymphocytes, and plasma cells in the polyp specimens typical of allergic reaction. The SNA and MAA I staining in polyp tissues were more intense and covered more cells than that observed in normal mucosal tissues. Both SNA and MAA I lectins stained mucosal surface of polyps, whereas normal nasal mucosa showed positive staining with SNA on the mucosal surface and positive staining with MAA I in submucosal glands (Figure 1a). In order to provide a quantitative measurement, we counted cells with positive staining on their apical surface. While 79.93±10.06% and 71.63±22.21% of epithelial cells on nasal polyps expressed α2,3- and α2,6-linked sialic acid, respectively, only 17.94±16.83% and 35.64±12.51% of epithelial cells on normal nasal mucosa expressed the receptors. These differences are statistically significant at p<0.01 for α2,3-linked sialic acid and p<0.05 for α2,6-linked sialic acid, by t-test. This indicates that the α2,3-linked sialic acid, which is the receptor for AIV, is present on mucosal surface of nasal polyps but not on normal nasal mucosa. The lectin staining pattern of normal nasal mucosa is in agreement with what has been previously reported [4]. In order to confirm the expression of α2,3-linked sialic acid on the nasal polyps, we digested fresh tissue of nasal polyp by 1U/ml sialidase before performing the lectin staining. The sialidase digestion eliminated the MAA I staining signal from the apical surface of the epithelial cells confirming the specificity. Staining in basal cells was not eliminated probably because they were not accessible to the enzyme as the digestion was done on an intact piece of tissue (Figure 1b).

### Infection of tissue explants by influenza viruses

**Figure 2:**
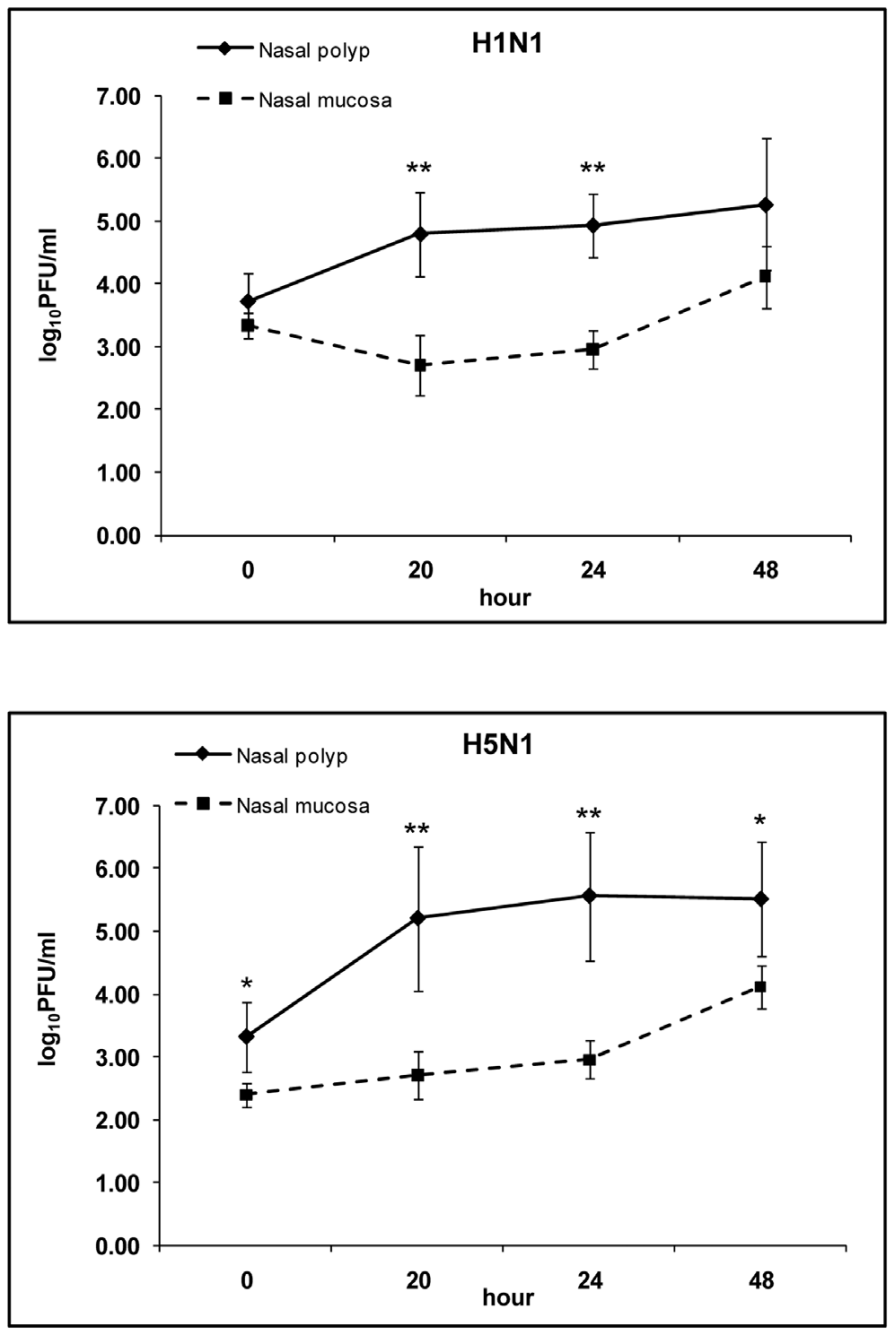
Infection of human and avian influenza viruses in nasal polyp and nasal turbinate mucosal explants. Two pieces of tissue of about 3–4 mm in diameter from each specimen were cultured in a 24-well tissue culture plate. Each tissue explant was infected with 1×10^6^ TCID50 of H5N1 AIV [A/Thailand/3 (SP-83)/04] or seasonal influenza virus H1N1 [A/Thailand/Siriraj-3/06]. After two hours infection the unattached virus was removed by washing twice with PBS. At 0, 20, 24 and 48 hr post-infection, the culture supernatants were collected for virus titrating by plaque assay. The data were derived from two nasal polyps and two nasal mucosa. One or two asterisks indicate a statistical significance at a *P* value of <0.05 or <0.01, respectively, as determined by a t test for a comparison between viral titers from nasal polyp and nasal mucosa at the same time point.

**Figure 3:**
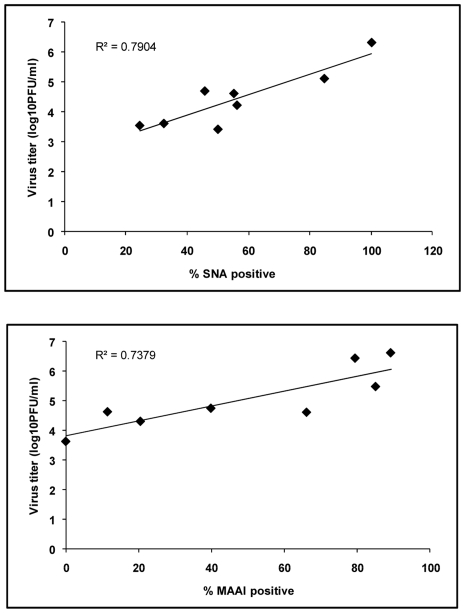
Correlation between the percentages of lectin-positive cells and the viral titers. Dot plots of percentages of lectin-positive cells versus maximum viral titers produced from the same tissue samples show linear correlation with Pearson correlation coefficient of 0.889 for SNA (p = 0.003) and 0.859 for MAA I (p = 0.006). The data were derived from the same experiments shown in Figure 1, 2 and 4.

To test whether the increased sialic acid expression on nasal polyps would result in enhanced susceptibility to influenza infection, tissue explants were infected with influenza viruses. Tissue explants were derived from 2 nasal polyps and 2 normal nasal mucosal tissue samples. Both the seasonal influenza virus [A/Thailand/Siriraj-3/06 (H1N1)] and the highly pathogenic AIV [A/Thailand/3 (SP-83)/04 (H5N1)] could infect normal nasal mucosa explants as indicated by the increase of viral titer in the culture supernatant. The titers of H5N1 AIV were comparable to or even somewhat higher than those of seasonal H1N1. This may reflect the higher replication efficiency of H5N1 as seen in various cell lines (data not shown). Interestingly, both the seasonal influenza and the H5N1 AIV replicated to higher titers in nasal polyp explants (Figure 2). The maximum viral titers showed correlation with the percentages of sialic acid-positive cells on the same tissue samples (Figure 3).

**Figure 4:**
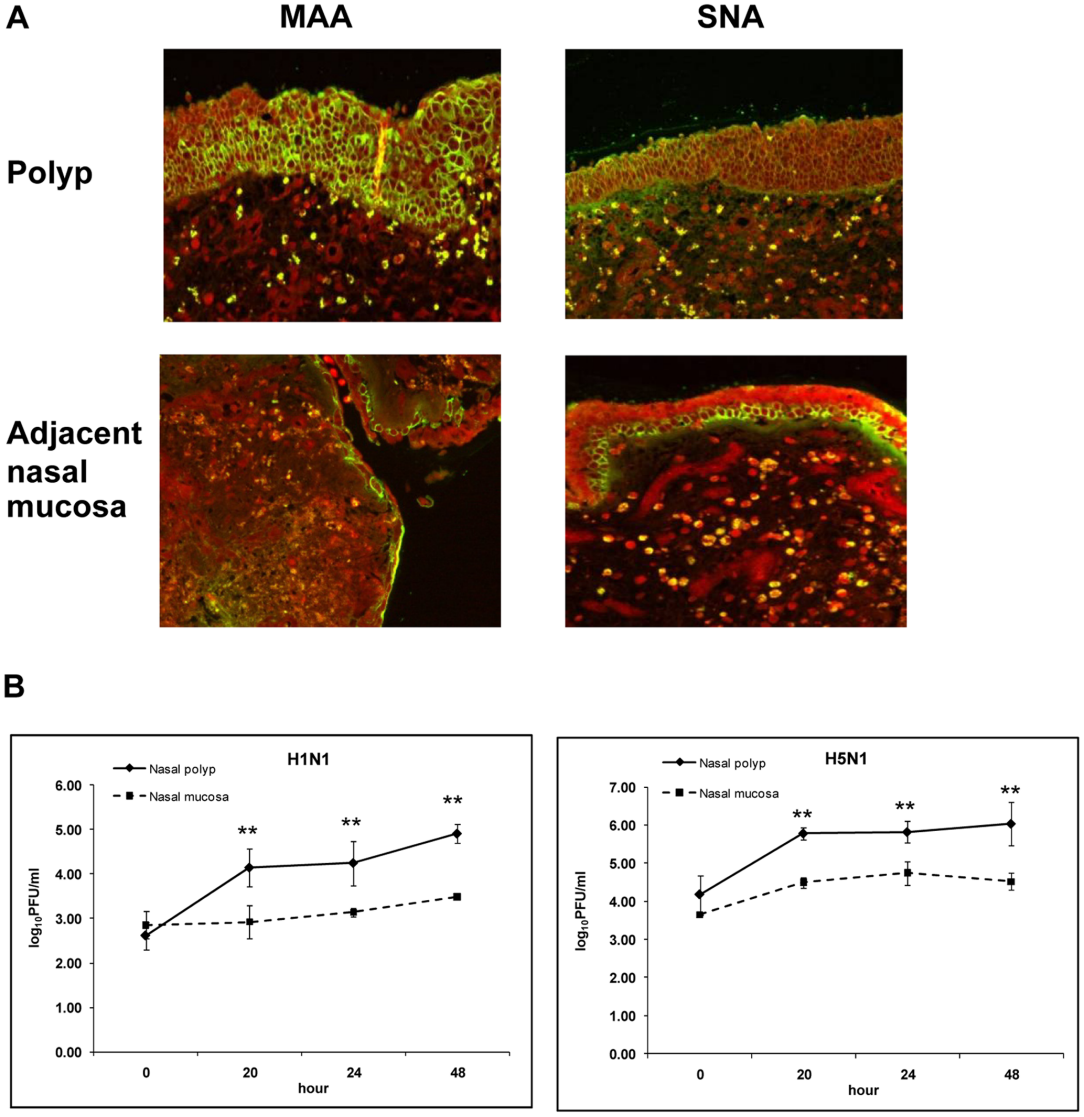
Distribution of receptor and infection of nasal polyps and adjacent normal mucosa. Distribution of sialic acid receptors (a) and outputs of viral infection (b) of tissue samples from nasal polyps and adjacent normal mucosa from the same patients. The data were derived from experiments using nasal polyps and adjacent normal nasal mucosal tissue samples from two patients.

To ensure that the difference between nasal polyps and normal nasal mucosa was not because of normal variation among individuals, we repeated the experiments using tissue samples from nasal polyps and adjacent normal mucosa from the same patients, and similar results were observed (Figure 4). This indicated that nasal polyps were indeed more efficiently infected by seasonal and avian influenza viruses.

**Figure 5:**
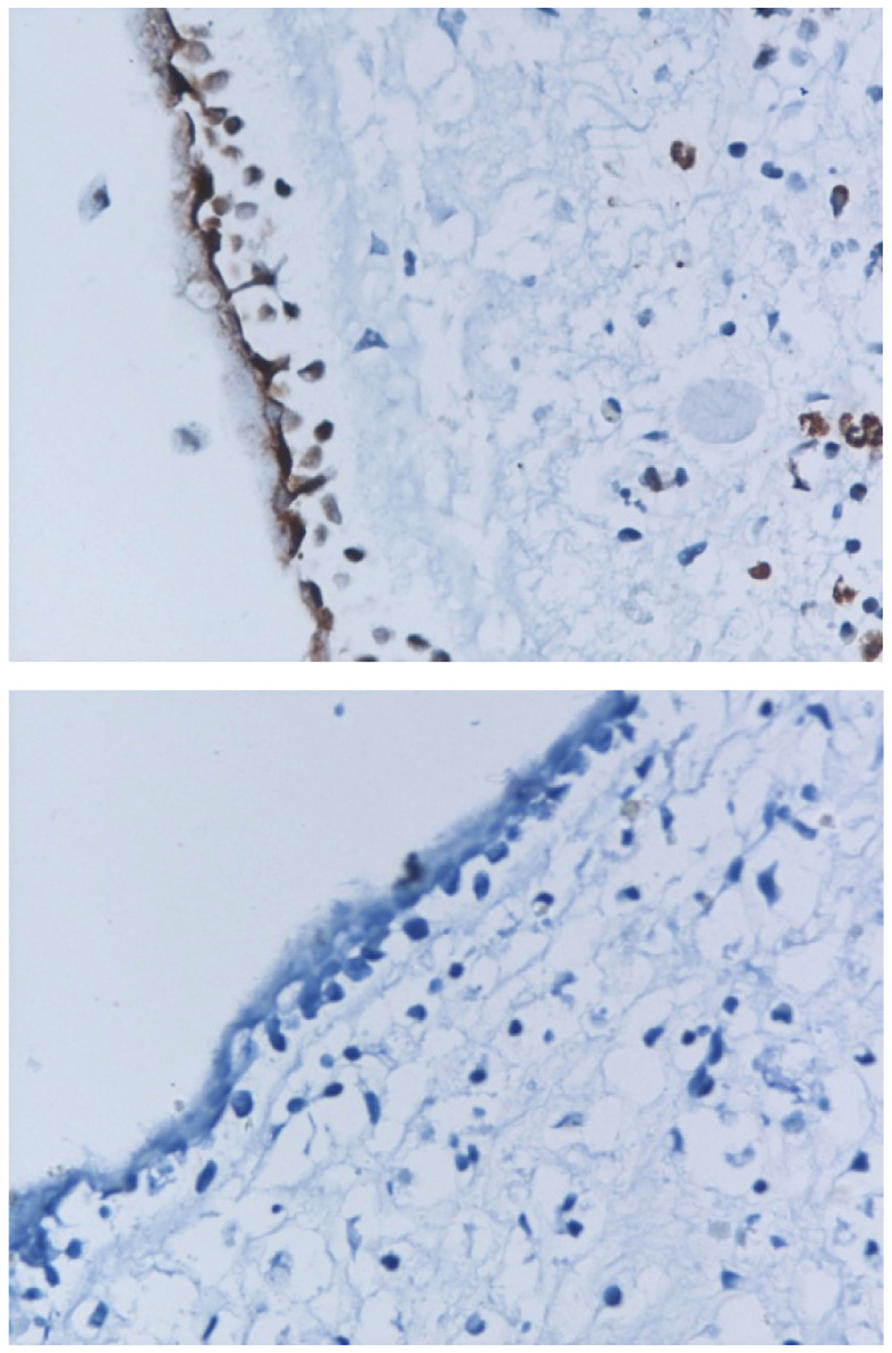
In situ hybridization of H5N1 AIV-infected (upper) and non-infected (lower) polyp tissues. The hybridization signal is shown in red-brown color in the epithelial cells on the mucosal surface of infected tissue.

We performed in situ hybridization on sections of infected and non-infected polyp tissues. Positive hybridization signal was observed on the mucosal surface of infected polyp, while the non-infected polyp was negative (Figure 5). This indicates that the infection occurred in the epithelial cells, where the viral receptor was present, and further supports the association between the presence of viral receptor and the infection.

### Expression of ST3GAL1 and ST3GAL4 mRNA

**Figure 6:**
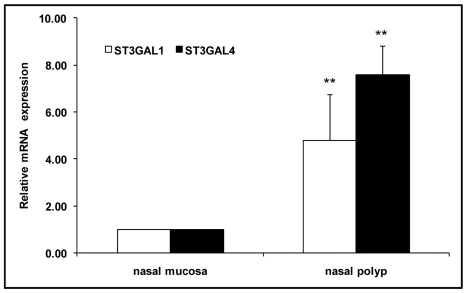
Expression of ST3GAL1 and ST3GAL4 mRNA measured by quantitative real time RT-PCR. The amount of total RNA was normalized using GAPDH mRNA. The data are shown as the mean ± SD from an experiment done in triplicate. The data were derived from two pieces of tissues from the same patient. The difference between mRNA expression in nasal polyp and nasal mucosa, marked by asterisks, is statistically significant (t test, *P*<0.01).

In order to explore the mechanism of sialic acid upregulation, we measured mRNA levels of ST3GAL1 and ST3GAL4, which are the major enzymes responsible for adding sialic acid to galactose through the α2,3-linkage [13]. Both of the enzymes showed higher levels of mRNA in nasal polyp (Figure 6). This suggests that up-regulation of the enzymes is responsible for the observed sialic acid up-regulation.

## Discussion

Previous reports showed that nasal polyp tissue explants could be infected by influenza viruses [14], [15]. The authors suggested that nasal polyp tissue can be used as an in vitro model for influenza infection. These reports are in agreement with our data showing more efficient infection in nasal polyp explants as compared to normal nasal mucosa. Furthermore, our data show a correlation between cell surface sialic acid availability and the efficiency of infection, suggesting that receptor availability may be a major determinant for efficient infection in respiratory epithelium.

In addition to sialic acid upregulation, allergic and inflammatory conditions can cause a number of changes including upregulation of various cytokines, such as IL-1, IL-4, IL-5, IL-8, and TNF-α [16]. Whether these changes contribute to the sialic acid upregulation and the increased efficiency of influenza infection is not clear. NF-κB is required for influenza virus replication [17], and it has been shown to be upregulated in nasal polyp [18]. It is possible that upregulation of NF-κB may also contribute to the enhanced influenza infection in the nasal polyp tissue.

H5N1 AIV transmission in humans is inefficient [19]. Only a small fraction of exposed individuals became infected [20]. Clusters of infected individuals are often within blood-related individuals [21], suggesting host factors contributing to the susceptibility to the infection. Variability of sialic acid expression on mucosa of upper respiratory tract is likely to be one of the factors. Here, we show that sialic acid on nasal mucosa and the efficiency of H5N1 AIV infection can be upregulated by allergic and inflammatory conditions and that the upregulated α2,3-linked sialic acid can support H5N1 AIV infection [22], [23], [24]. This suggests that conditions capable of upregulating α2,3-linked sialic acid in human upper airway may contribute to susceptibility to H5N1 AIV infection in exposed individuals. This variation in sialic acid expression may provide an opportunity for AIVs to infect certain individuals as entry points into human population, which provide a chance for further adaptation, which will enable the virus to gain full access into human population. Understanding this may be crucial for preventing emergence of a pandemic virus.

Field evidences linking nasal polyp and increased influenza susceptibility are lacking. However, allergic conditions such as asthma, which is associated with allergic nasal conditions, have been shown to be associated with upper respiratory tract infection [22], [23], [24]. Most investigators consider this relationship as triggering of asthmatic attack by viral infection. Nevertheless, the association exists and whether allergic persons are more susceptible to viral infection or influenza has not been fully explored. As for nasal allergy, the signs and symptoms of the allergy can be confused with those of viral infection, which further complicates the observation of association between the two conditions. Carefully designed clinical studies are needed to explore this possible relationship.

Although our data are derived from in vitro experiments, our experimental system involves only minimal in vitro handling of the tissue and is likely to resemble what could happen in vivo. Nevertheless, whether incidence of seasonal influenza and the risk of H5N1 AIV infection in exposed individuals can be indeed influenced by allergic conditions and levels of cell surface sialic acid requires further exploration and clinical investigations.
